# Integrative analysis of fungal communities in three types of Baijiu Daqu using third-generation sequencing and culturomics

**DOI:** 10.3389/fmicb.2025.1748163

**Published:** 2026-01-14

**Authors:** Yu-Hua Wei, Da-Yong Han, Liang Song, Liang-Chen Guo, Mei Bai, Pei-Jie Han, Hai-Yan Zhu, Zhang Wen

**Affiliations:** 1 State Key Laboratory of Microbial Diversity and Innovative Utilization, Institute of Microbiology, Chinese Academy of Sciences, Beijing, China; 2 College of Life Sciences, University of Chinese Academy of Sciences, Beijing, China; 3 College of Life Science, University of Hebei, Baoding, China; 4 GuiZhou XiJiu Co., Ltd., Guizhou, China

**Keywords:** culturomics, Daqu, diversity, fungal communities, third-generation sequencing

## Abstract

Daqu, the core fermentation starter for Chinese Baijiu, harbors intricate and functionally important fungal communities. To comprehensively characterize its biodiversity, we employed an integrated approach combining third-generation amplicon sequencing with culturomics to systematically investigate the fungal communities in low-, medium-, and high-temperature Daqu. Our analysis identified 109 amplicon sequence variants and 135 cultivable isolates, encompassing both cultivable and non-cultivable fungal taxa. Third-generation sequencing revealed greater fungal species richness, whereas culturomics effectively isolated dominant functional fungi, including *Saccharomycopsis fibuligera* and *Lichtheimia ramosa*. Moreover, the fungal communities in Daqu exhibited distinct temperature-dependent patterns, with the thermophilic fungus *Thermoascus crustaceus* being the predominant species in high-temperature Daqu. This integrative approach provides complementary insights into the fungal ecology of Daqu and establishes a foundation for the development fungal resources with potential industrial applications.

## 1 Introduction

As one of the world’s six major distilled spirits and among the oldest strong alcoholic beverages, Chinese Baijiu has exerted a profound impact on society, economy, and politics (Jin et al., 2017). According to the China Alcoholic Drinks Association, Baijiu production reached 4.14 million tons in 2024, generating industry sales revenue of 796.3 billion yuan. The Baijiu production process comprises two main stages: Daqu fermentation and Baijiu fermentation (Wen et al., 2024). Based on the maximum fermentation temperature of Daqu, it is categorized into low-temperature Daqu (LTD), medium-temperature Daqu (MTD) and high-temperature Daqu (HTD), which are employed to produce light-, strong-, and sauce-flavor Baijiu, respectively (Sakandar et al., 2020). Daqu is produced through solid state fermentation using wheat as the primary raw material in a natural, open environment, inherently resulting in a complex microbial consortium that play a crucial role in grain saccharification during Baijiu fermentation and in contributing aromatic compounds to the final product (Han et al., 2024).

The microbial community in Daqu comprises diverse yeasts, filamentous fungi and bacteria (Xia et al., 2023). Notably, previous studies have demonstrated that Daqu serves as the primary source of fungi for Baijiu fermentation (Wang et al., 2018; Wu et al., 2021). Among these microorganisms, filamentous fungi can produce a wide range of enzymes that degrade proteins, starch and other macromolecular substances, thereby providing essential saccharification power and enzymatic activity for Daqu production and Baijiu fermentation. Gan et al. (2019) revealed the important contribution of filamentous fungi species from the order *Eurotiales* in promoting the saccharification process in MTD and HTD. Bai et al. (2024) through metaproteomic and culture-dependent analysis, found that *α*-amylase and glucoamylase from *Aspergillus* and *Rhizopus* dominated the bio-system of MTD. In addition, yeasts are responsible for the production of alcohols, esters, and various flavor metabolites. Among them, *Saccharomyces cerevisiae* is considered the most important yeast species for alcoholic fermentation due to its ability to ferment various sugars into ethanol (Li et al., 2018). Meanwhile, *non-Saccharomyces* yeasts from Daqu play a crucial role in producing complex aromatic compounds that enhance the overall flavor profile of Baijiu (He et al., 2022). For example, species of the genus *Pichia* are among the most important functional yeasts in Baijiu fermentation, capable of utilizing sucrose and glucose to produce various aromatic compounds, including ethanol, ethyl acetate, and 4-hydroxy-2-butanone (Li et al., 2011, 2016). Therefore, the fungal communities within Daqu play a crucial role in determining both the quality of Daqu and the Baijiu fermentation process.

The diversity and function of fungal communities in Daqu have become a research hotspot in the field of Baijiu microbiology (Xu et al., 2017; Wang D. et al., 2019). Early studies on fungal communities primarily focused on isolating and purifying strains using traditional culture-dependent methods (Zou et al., 2018; Zhang et al., 2024). However, the microbial communities in Daqu and its brewing environment are highly complex and diverse, and traditional culture-dependent techniques can capture only 0.1–1.0% of the microorganisms present, greatly limiting our understanding of fungal diversity in Chinese Baijiu fermentation (Kang et al., 2022; Li et al., 2023). With advances in sequencing technologies, next-generation sequencing (NGS) and third-generation sequencing (TGS) have been increasingly applied to investigate the Daqu microbiota (Han et al., 2024; Wen et al., 2024, 2025). Compared with NGS, TGS enables full-length amplicon sequencing, thereby providing higher taxonomic resolution and improved accuracy in species-level identification (Guo et al., 2026). Nevertheless, amplicon-based sequencing cannot generate live isolates, which limits downstream functional validation and the industrial exploitation of key strains (Fan et al., 2019, 2020; Chen et al., 2021).

In this background, the integrated application of multi-omics approaches has provided a new research paradigm for system-level investigations of complex biological systems (Ferrocino et al., 2023). This systems biology-oriented strategy has gradually evolved into the concept of ‘Qu-omics’, which refers to the comprehensive investigation of Daqu quality, functionality, manufacturing processes, and fermentation performance through the integration of multiple omics technologies (Yang et al., 2024). Nevertheless, despite the continuous advancement of Qu-omics research, most existing studies still rely heavily on sequencing-based community profiling and large-scale omics datasets (Mu et al., 2025; Xie et al., 2026). Consequently, high-resolution species-level identification and the systematic recovery of viable microbial resources remain relatively insufficient, particularly with respect to fungal resources required for functional validation and industrial applications.

Culturomics is a recently developed strategy for exploring complex microbial ecosystems and has achieved notable success in studies of the human gut microbiome (Naud et al., 2020; Mukhopadhya et al., 2022). By integrating diverse and optimized culture conditions, culturomics can overcome some of the limitations of traditional culture-dependent techniques and sequencing-based approaches, including TGS (Fournier et al., 2015). Therefore, within the Qu-omics research, the combination of TGS and culturomics represents a complementary strategy that not only enhances the resolution of fungal diversity analysis but also facilitates the systematic establishment of fungal resources for downstream functional studies and industrial exploitation.

In this study, TGS and culturomics were integrated to systematically analyze the fungal communities in LTD, MTD, and HTD. This combined approach aimed to compare the differences between the two methods in revealing fungal diversity and community structure, to identify representative fungal taxa characteristic of each Daqu type, and to evaluate the complementarity of TGS and culturomics in uncovering fungal diversity. These findings are expected to broaden our understanding of fungal diversity in Baijiu fermentation and provide a scientific basis for fungal resource development and the improvement of Daqu quality.

## 2 Materials and methods

### 2.1 Sample collection

**Figure 1:**
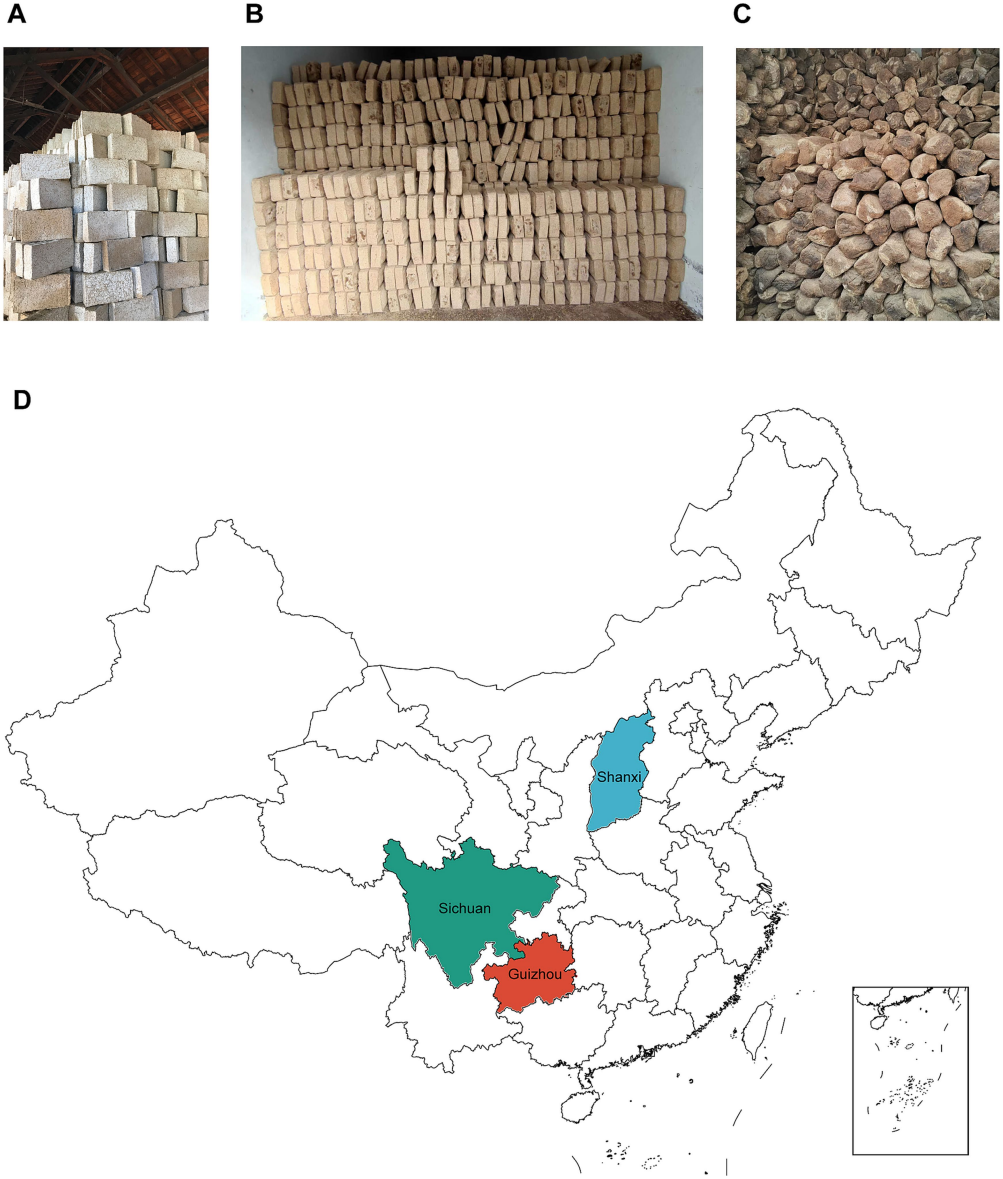
Mature low-temperature Daqu **(A)**, medium-temperature Daqu **(B)**, and high-temperature Daqu **(C)** in the storage room. Geographic distribution of sampling sites for the three types of Daqu **(D)**.

LTD (Figure 1A), MTD (Figure 1B), and HTD (Figure 1C) samples were obtained from three representative Baijiu enterprises located in the core production regions of Shanxi, Sichuan, and Guizhou provinces, China, respectively (Figure 1D). These enterprises were selected for their long-term stable production practices and representativeness of typical Daqu production processes. In October 2023, mature Daqu bricks that had undergone natural fermentation and were stored for 3 months were randomly collected from the Daqu storage rooms of each enterprise. Three Daqu bricks were obtained from each site, yielding a total of nine samples. After collection, all samples were thoroughly ground, homogenized, and subdivided into sterile polyethylene bags. Each sample was divided into two portions: one was immediately subjected to microbial isolation and cultivation, while the other was stored at −80 °C for subsequent genomic DNA extraction and TGS (PacBio platform).

### 2.2 Isolation, cultivation, and purification of fungal strains

To obtain a comprehensive representation of fungal diversity, six types of culture media were employed: potato dextrose agar (PDA; 20% potato infusion, 2% glucose and 2% agar), PDA supplemented with 1% Daqu extract, bengal red agar (1% peptone, 2% glucose, 0.1% dipotassium phosphate, 0.005% magnesium sulfate, 0.0033% rose bengal and 2% agar), G25N agar (25% glycerol, 0.37% yeast extract, 0.10% dipotassium phosphate, 0.225% sodium salt, 0.0375% potassium chloride, 0.0375% magnesium sulfate heptahydrate, 0.00075% ferrous sulfate heptahydrate and 2% agar), yeast extract-peptone-dextrose agar (YPD; 1% yeast extract, 2% peptone, 2% glucose and 2% agar), and YPD supplemented with 0.2% (v/v) acetic acid. Microbial isolation and cultivation were performed using the conventional dilution plating method. Samples were initially subjected to a tenfold dilution and vortexed for 5 min to achieve thorough homogenization. Serial tenfold dilutions were then prepared up to 10^−5^. Aliquots of 100 μL from four dilution levels (10^−2^, 10^−3^, 10^−4^, and 10^−5^) were spread onto six different media to maximize the recovery of diverse fungal isolates. PDA was incubated at 45 °C for the isolation of thermophilic filamentous fungi (Han et al., 2023). PDA supplemented with 1% Daqu extract and Bengal red agar were incubated at 30 °C for the isolation of mesophilic filamentous fungi (Li et al., 2022). G25N agar was used at 30 °C to isolate osmotolerant fungi (Pitt, 1973). YPD and acidified YPD (YPD + 0.2% acetic acid) were incubated at 30 °C for yeast isolation, with the acidified medium effectively suppressing filamentous fungi growth (Guan et al., 2019). The Daqu extract for PDA enrichment was prepared by boiling 10 g of crushed Daqu in 600 mL of distilled water, followed by filtration and volume adjustment to 1 L with distilled water to provide trace nutrients. All media contained 2% (w/v) agar and were sterilized at 115 °C for 30 min. After cooling to approximately 55 °C, chloramphenicol (0.2%, v/v; 100 mg/mL stock solution) was added to inhibit bacterial growth. The inoculated plates were incubated in an inverted position at the corresponding temperatures for 24–72 h until visible colonies appeared. Representative colonies were selected based on morphological characteristics and subcultured on the corresponding medium to obtain pure isolates. The purified strains were confirmed by streak plating and stored in 25% (v/v) sterile glycerol at −80 °C for long-term preservation.

### 2.3 Identification of fungal strains

Yeast genomic DNA was extracted following the method described by Wei et al. (2025a), while genomic DNA from filamentous fungi was extracted using a plant DNA magnetic bead extraction kit (Geneonbio, Changchun, China). The quality and concentration of the extracted DNA were assessed using 1% (w/v) agarose gel electrophoresis and a NanoDrop spectrophotometer (Thermo Scientific, USA). To identify yeast species, the D1/D2 region of 26S rDNA was amplified using the primers NL1 (5′-GCA TAT CGG TAA GCG GAG GAA AAG-3′) and NL4 (5′-GGT CCG TGT TTC AAG ACG G-3′) (O’donnell, 1993), while the ITS region was amplified using the primers ITS1 (5′-TCC GTA GGT GAA CCT GCG G-3′) and ITS4 (5′-TCC TCC GCT TAT TGA TAT GC-3′) for filamentous fungi identification (White et al., 1990). The amplification reaction volume was 25 μL, consisting of 22 μL of 1.1 × T3 Super PCR Mix (Tsingke Biotech Co., Beijing, China), 1 μL of DNA template, and 1 μL of each primer. The PCR cycling conditions were as follows: initial denaturation at 98 °C for 2 min; followed by 35 cycles of denaturation at 98 °C for 10 s, annealing at 55 °C for 15 s, and extension at 72 °C for 15 s; with a final extension at 72 °C for 5 min. To accurately identify certain filamentous fungi, the calmodulin gene (CaM) was amplified using the primers CMD5 (5′-CCG AGT ACA AGG ARG CCT TC-3′) and CMD6 (5′-CCG ATR GAG GTC ATR ACG TGG-3′) (Samson et al., 2014), and the *β*-tubulin gene was amplified using the primers Bt2a (5′-GGT AAC CAA ATC GGT GCT GCT TTC-3′) and Bt2b (5′-ACC CTC AGT GTA GTG ACC CTT GGC-3′) (Hubka and Kolarik, 2012). These amplifications were performed using the same reaction volume and PCR cycling conditions as described above. PCR products were visualized by electrophoresis on a 1% (w/v) agarose gel under UV light. The single band of the expected size was directly sequenced by Bio Magic Gene Company.^1^ Sequences were analyzed using Chromas Lite v.2.1 and compared to the GenBank nucleotide database using BLAST.^2^

To complement the molecular identification results, representative strains of the dominant fungal species were subjected to systematic morphological characterization. Macroscopic colony features on individual plates were documented using a digital camera (Canon EOS 90D, Japan) or a stereomicroscope (Nikon SMZ25, Japan). For microscopic observations, high-resolution images of cells or hyphae were obtained using a scanning electron microscope (Zeiss Axio Imager A2, USA). All morphological features were compared with standard taxonomic references for yeasts (Kurtzman et al., 2011) and filamentous fungi (Pitt and Hocking, 2022) to validate the molecular identification results.

### 2.4 DNA extraction, PCR amplification and third-generation sequencing

The total Genomic DNA was extracted from samples following a modified method described by Luo et al. (2023), with the tissue grinding step performed at 60 Hz for 2 min to ensure thorough cell disruption. DNA purity was evaluated by measuring the A260/A280 ratio using a NanoDrop™ One spectrophotometer (Thermo Fisher Scientific, USA), and DNA integrity was assessed by 1% (w/v) agarose gel electrophoresis. DNA samples were normalized to an equal concentration using nuclease-free water prior to PCR amplification. The full-length fungal ITS region was amplified using primers ITS1 and ITS4, with PCR conditions and cycling parameters as described by Wei et al. (2025b). Positive and negative controls were included to ensure amplification reliability, and no amplification or detectable contamination was observed in the negative controls. PCR products were purified with AMPure PB magnetic beads (Beckman Coulter, USA) to remove residual primers and impurities. Library preparation and sequencing were performed by Annoroad Gene Technology Co., Ltd. (Beijing, China) on the PacBio Sequel II platform.

### 2.5 Sequencing data processing

PacBio amplicon sequencing data were processed following the standard SMRT analysis workflow to ensure the generation of high-quality sequences. Initially, barcode sequences of each sample were identified and demultiplexed using Lima v1.7.0 to obtain circular consensus sequences (CCS). Primer sequences were subsequently removed with Cutadapt v2.7 (Kechin et al., 2017), and only ITS fragments within the 300–1,000 bp length range were retained. To further improve sequence accuracy, fungal chimeric reads were identified and removed using the UNITE CHIME reference database (release October 16, 2022) (Nilsson et al., 2019). Subsequently, the unique sequences were denoised using the UNOISE3 algorithm to generate amplicon sequence variants (ASVs) with 100% nucleotide identity, with the minsize parameter set to 10. The resulting ASVs were taxonomically assigned by comparison against the UNITE UTAX reference dataset (released July 25, 2023) (Nilsson et al., 2019), using a 97% sequence similarity threshold as the criterion for species-level annotation. To further support taxonomic assignments, representative ASVs with high relative abundance were cross-validated using BLASTn searches against the NCBI nucleotide database.

### 2.6 Statistical analysis and visualization

The sequences obtained by Sanger sequencing were aligned with those of type strains from related species using MEGA v.7 (Kumar et al., 2016). A phylogenetic tree was subsequently constructed using the Neighbor-Joining (NJ) method in MEGA v.7 with 1,000 bootstrap replicates (Kumar et al., 2016; Lachance, 2022). The resulting phylogenetic tree was visualized using the Interactive Tree of Life (iTOL) online platform (Letunic and Bork, 2016). To ensure comparability among samples, the ASV table was rarefied to the minimum sequencing depth (3,052 reads per sample) using the rrarefy function. Subsequently, the fungal community composition and diversity were analyzed in R (v4.3.2) using the ggplot2, vegan, dplyr, and igraph packages. Alpha diversity indices (ACE, Chao1, Richness, and Shannon) were calculated using the diversity function, and differences among groups were assessed by analysis of variance (ANOVA) followed by Tukey’s HSD test. Furthermore, differential taxa were further identified using the MicrobiotaProcess package following the LEfSe statistical framework, which applies Kruskal–Wallis and Wilcoxon rank-sum tests; taxa with an LDA score > 2.0 were considered significant biomarkers. The results were visualized with ggtree and ggplot2 packages. In addition, shared and unique taxa among groups were visualized using Upset, Venn, and Venn network plots generated by the UpSetR, VennDiagram, and igraph packages, respectively. Macroscopic and microscopic images of the dominant fungal species were arranged and compiled using Adobe Illustrator 2020.

## 3 Results

### 3.1 Culturomics based characterization of fungal communities in three types of Daqu

**Figure 2:**
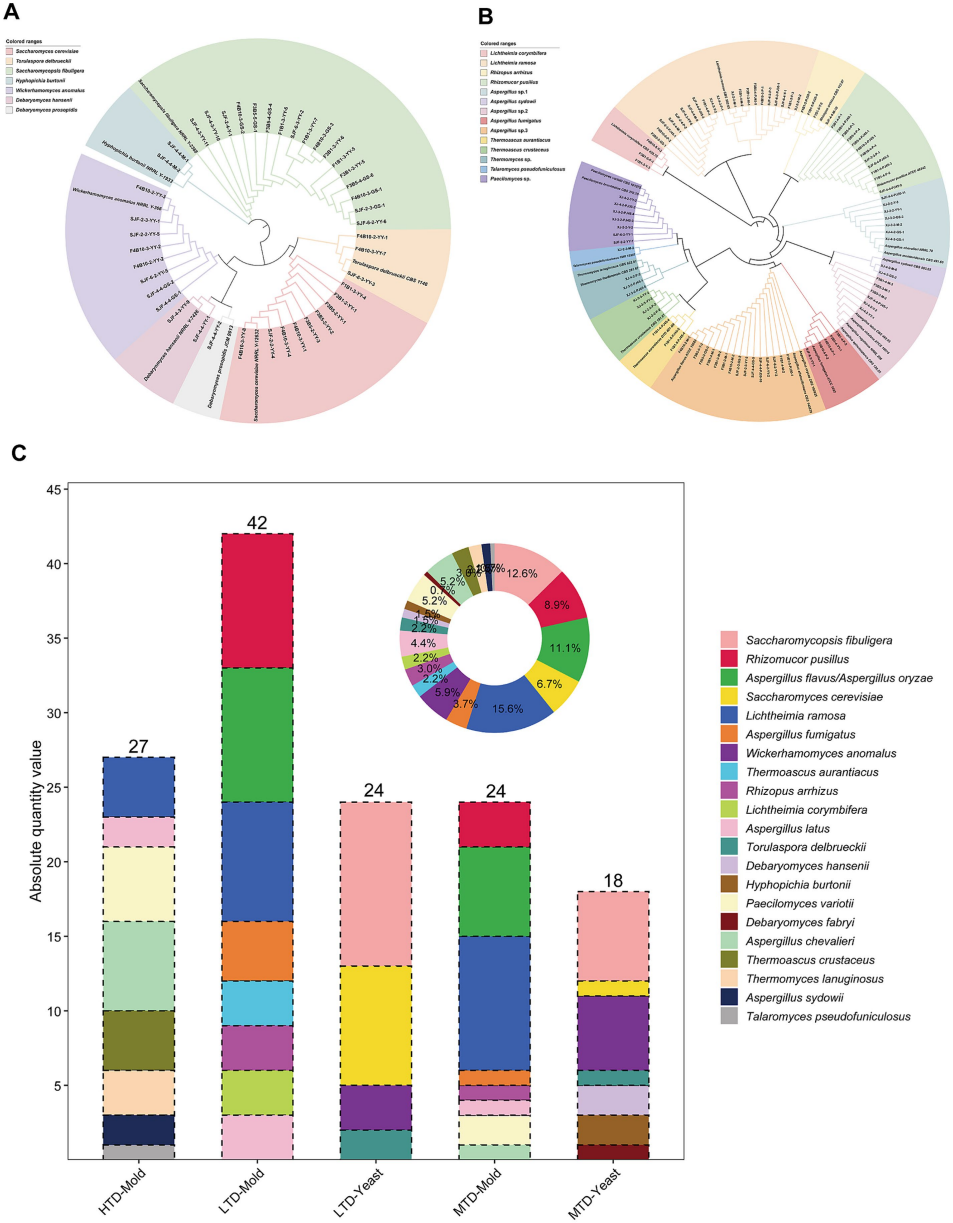
Culturomics-based analysis revealing the phylogenetic relationships and isolation profiles of culturable yeasts and filamentous fungi in three types of Daqu. **(A)** Phylogenetic tree of yeasts constructed based on 26S rRNA gene sequences; **(B)** Phylogenetic tree of filamentous fungi constructed based on ITS sequences; **(C)** Number of isolates of each fungal species obtained from the three types of Daqu.

A total of 135 fungal isolates were obtained from the three types of Daqu based on culturomics, including 42 yeasts and 93 filamentous fungi. Phylogenetic analysis based on 26S rDNA sequences revealed that the yeast isolates belonged to six genera and seven species (Figure 2A), among which *Saccharomycopsis fibuligera* (12.6%, Supplementary Figure S1A), *S. cerevisiae* (6.7%, Supplementary Figure S1B), and *Wickerhamomyces anomalus* (5.9%, Supplementary Figure S1C) were the predominant species (Figure 2C). Meanwhile, ITS sequence-based phylogenetic analysis indicated that the filamentous fungi were classified into eight genera and 14 species (Figure 2B). Among these, five species within the genera *Aspergillus*, *Thermomyces*, and *Paecilomyces* could not be reliably resolved at the species level based solely on ITS sequences. Subsequent phylogenetic analyses using calmodulin (Supplementary Figure S2A) and *β*-tubulin (Supplementary Figure S2B) gene sequences further identified these isolates as *Aspergillus chevalieri*, *A. latus*, *A. flavus*/*oryzae*, *Thermomyces lanuginosus*, and *Paecilomyces* var*iotii*. Overall, the dominant filamentous fungi across the three Daqu types were *Lichtheimia ramosa* (15.6%, Supplementary Figure S1E), *A. flavus*/*oryzae* (11.1%), *Rhizomucor pusillus* (8.9%, Supplementary Figure S1D) and *Aspergillus fumigatus* (4.4%, Supplementary Figure S1F) (Figure 2C). Comparative analysis of fungal composition among the three types of Daqu showed that filamentous fungi were most abundant in LTD, dominated by *R. pusillus*, followed by HTD, dominated by *A. chevalieri*; and least abundant in MTD, where *L. ramosa* was the major species. Yeasts were most frequently isolated from LTD and to a lesser extent from MTD, with *S. fibuligera* as the dominant species in both, whereas no culturable yeast was recovered from HTD.

### 3.2 TGS based characterization of fungal communities in three types of Daqu

**Figure 3:**
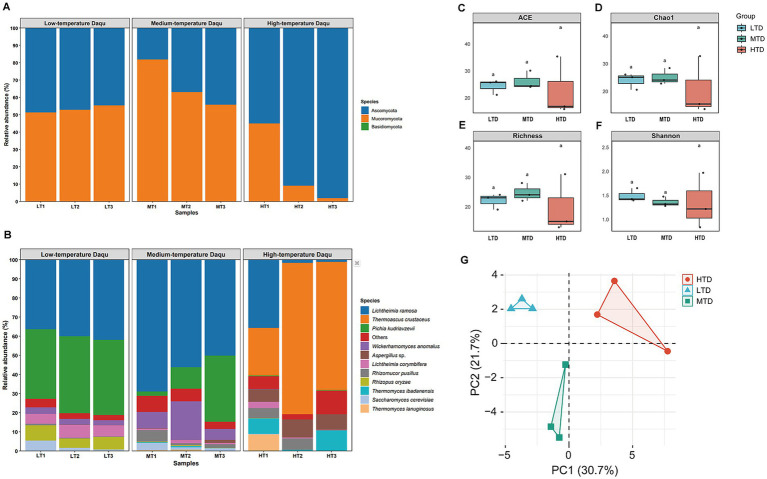
Relative abundance of fungal community in low-temperature Daqu (LTD), medium-temperature Daqu (MTD), and high-temperature Daqu (HTD) at the phylum **(A)** and species **(B)** levels. Species with a relative abundance lower than 0.5% in all samples were classified as “others.” Comparison of *α*-diversity indices, including ACE **(C)**, Chao1 **(D)**, Richness **(E)**, and Shannon **(F)** indices, among the three types of Daqu. Significant differences are indicated by different lowercase letters (*p* < 0.05). Principal component analysis (PCA) showing the overall differentiation of fungal communities in LTD, MTD, and HTD **(G)**. In panels C–G, LTD, MTD, and HTD are represented in blue, green, and red, respectively.

In this study, fungal DNA from three types of Daqu was analyzed using full-length ITS sequences. After quality filtering, a total of 61,890 high-quality ITS sequence pairs were obtained, with an average of 6,876 reads per sample. These sequences were classified into 109 fungal ASVs, representing to three phyla, 33 genera, and 54 species. Overall, Ascomycota (54%) and Mucoromycota (44%) were the dominant phyla, with Mucoromycota prevailing in MTD, Ascomycota predominating in HTD, and LTD exhibiting an intermediate distribution pattern (Figure 3A). At the species level, *L. ramosa* and *Pichia kudriavzevii* were dominant in LTD and MTD, whereas *Thermoascus crustaceus* was the predominant species in HTD (Figure 3B). Alpha diversity indices were applied to comprehensively assess the richness and evenness of the fungal communities (Herrera et al., 2023). The ACE and Chao1 indices were used to estimate species richness, with ACE emphasizing differences in abundance between rare and common taxa, and Chao1 estimating the number of unobserved rare taxa (Deng et al., 2021). The Richness index reflects the total number of detected species, while the Shannon index incorporates both abundance and evenness, representing overall community diversity (Supriatna, 2018). Comparative analysis among the three types of Daqu revealed that LTD exhibited the highest ACE, Chao1, and Shannon indices, MTD showed the highest Richness index, whereas HTD displayed the lowest values across all four indices, although these differences were not statistically significant (Figures 3C–F). Principal component analysis (PCA) further demonstrated clear separation of fungal community compositions among the three Daqu types, with PC1 and PC2 explaining 52.4% of the total variance. Notably, the LTD samples showed the least within-group variation, whereas HTD exhibited the greatest dispersion (Figure 3G).

### 3.3 Identification of differential fungal biomarkers among three types of Daqu based on TGS

**Figure 4:**
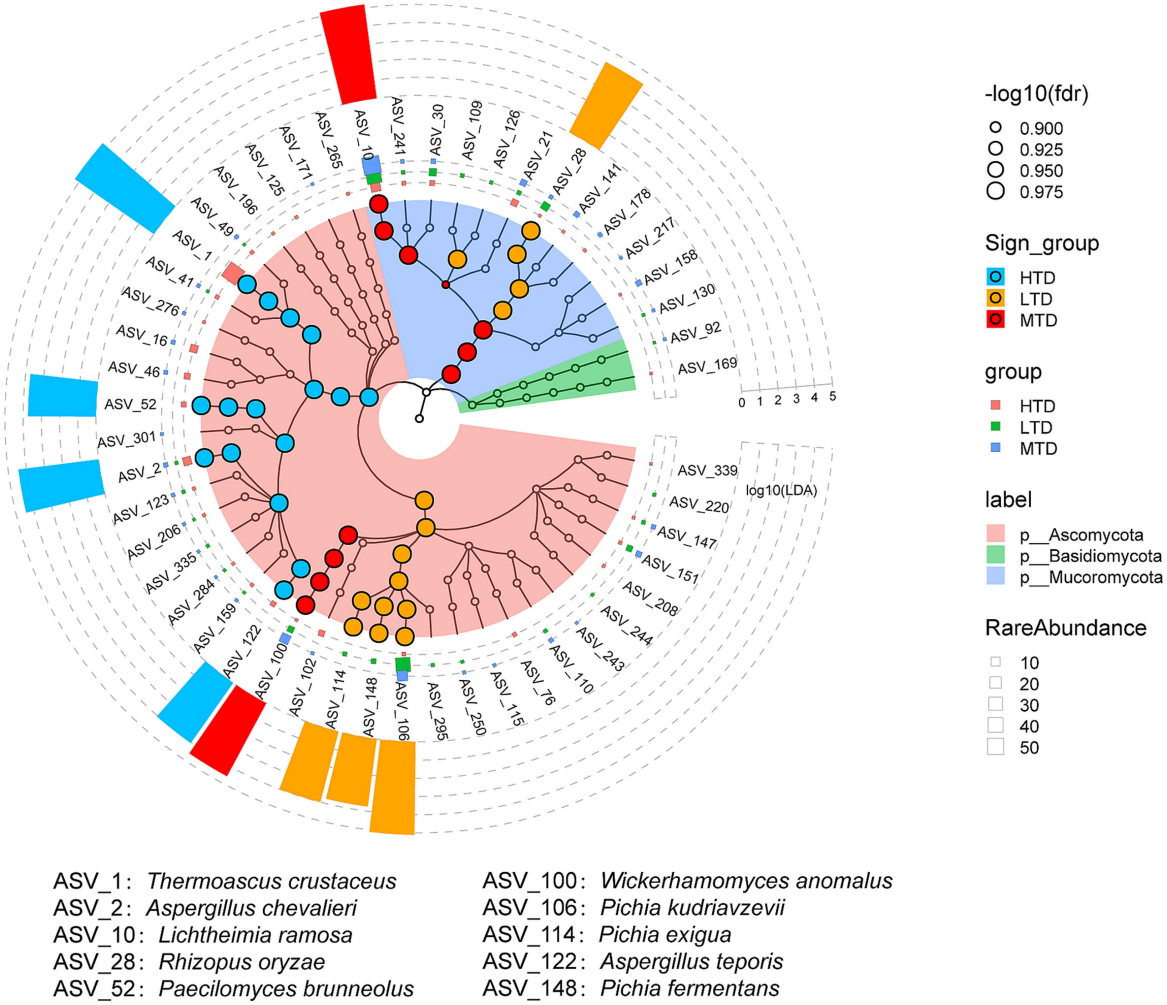
Identification of differential fungal taxa among the three types of Daqu using LEfSe analysis (LDA > 2, *p* < 0.05). The discriminant fungal taxa are shown in cladograms. Significant discriminant taxa for HTD, LTD, and MTD are represented in red, green, and blue, respectively, while non-discriminant taxa are shown in their corresponding phylum-level colors: Ascomycota in pink, Basidiomycota in light green, and Mucoromycota in light blue.

To elucidate differences in fungal community characteristics among the three types of Daqu, Linear discriminant analysis Effect Size (LEfSe) analysis was conducted based on third-generation sequencing data to identify potential fungal biomarkers (Figure 4). The results showed that four specific fungal taxa were identified in both LTD and HTD. In LTD, the identified biomarkers included *Rhizopus oryzae*, *P. kudriavzevii*, *Pichia exigua*, and *Pichia fermentans*, whereas in HTD they were *T. crustaceus*, *A. chevalieri*, *P. brunneolus*, and *Aspergillus teporis*. By comparison, MTD exhibited the fewest characteristic taxa, with only *L. ramosa* and *W. anomalus* detected as biomarkers. These findings highlight distinct fungal community compositions among the three Daqu types, providing insights into the fungal ecological characteristics shaped under different fermentation conditions.

### 3.4 Differences in fungal communities between TGS and culturomics in the same Daqu type

**Figure 5:**
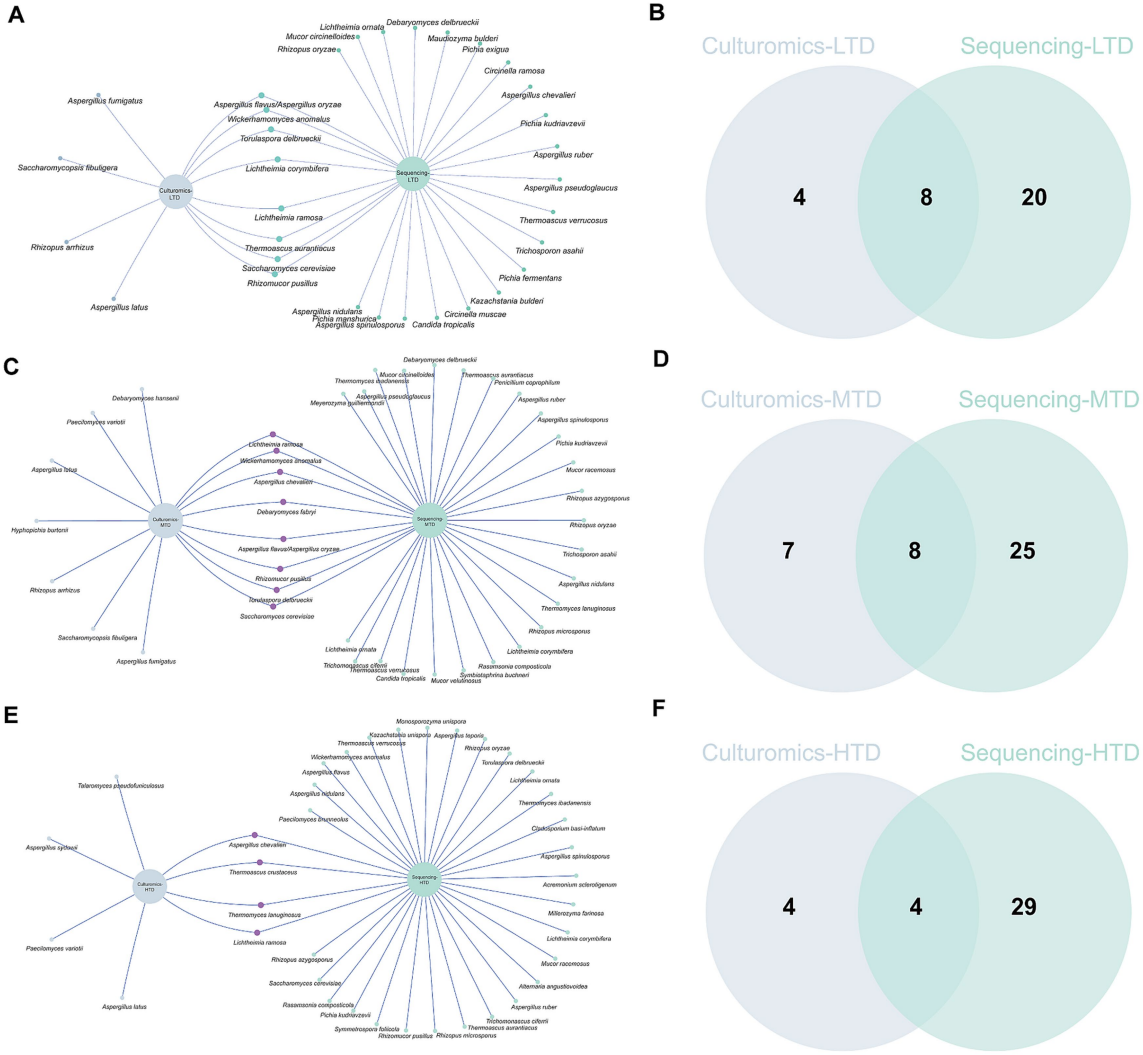
Fungal community composition and diversity in the same type of Daqu were compared using third-generation sequencing and culturomics: **(A,B)** LTD, **(C,D)** MTD, and **(E,F)** HTD.

To further explore the complementarity between sequencing- and culture-based approaches, we compared the fungal communities obtained by both methods across each Daqu type. The results revealed pronounced differences in fungal community structures among the different types of Daqu. Overall, TGS detected greater fungal richness, capturing a higher number of uncultured or difficult to culture taxa, whereas culturomics primarily recovered a limited set of cultivable dominant fungi. Specifically, TGS identified the largest number of characteristic species in HTD (29 species), while culturomics detected the most in MTD (seven species). Both approaches shared eight, eight, and four fungal species in LTD, MTD, and HTD, respectively (Figures 5B,D,F). Notably, *L. ramosa* and *T. crustaceus* were consistently detected as dominant species across all three types of Daqu by both methods, indicating their high ecological adaptability to varying temperature conditions (Figures 5A,C,E). In contrast, several dominant species were detected exclusively by only one of the two approaches. For instance, *S. fibuligera* and *P. variotii* were isolated exclusively through culturomics, whereas *P. kudriavzevii* was detected solely by TGS (Figures 5A,C,E). These differences suggest that integration both methods provide a more comprehensive understanding of the fungal community composition across different types of Daqu.

### 3.5 Differences in fungal communities among three Daqu types using TGS or culturomics

**Figure 6:**
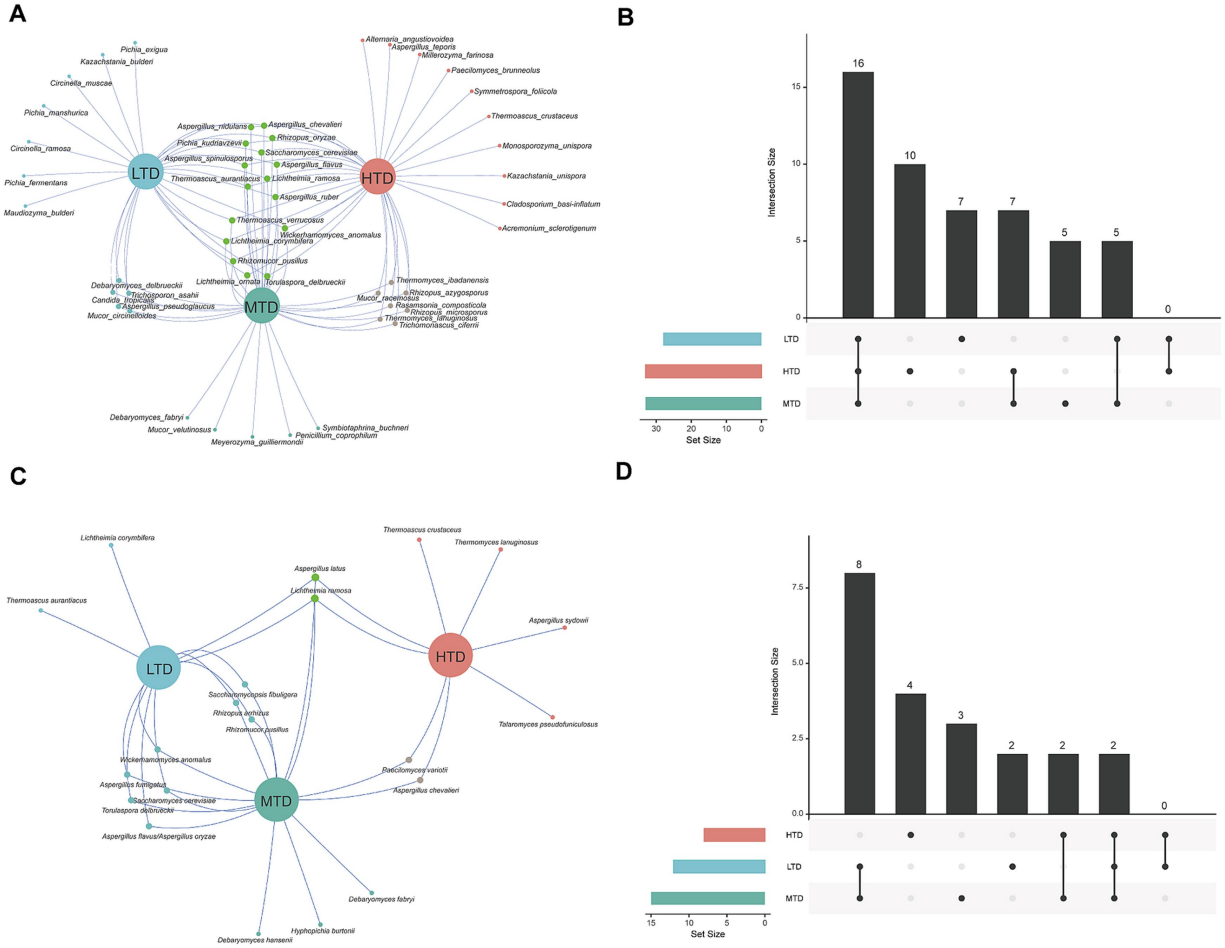
Comparison of fungal community composition and diversity among the three types of Daqu based on third-generation sequencing **(A,B)** or culturomics **(C,D)**.

The composition and diversity of fungal communities in three types of Daqu were systematically compared using TGS and culturomics. The results revealed significant differences in fungal community structures among the different Daqu types, with both methods exhibiting a degree of consistency and complementarity. Based on the TGS results, 16 fungal species were shared across all three Daqu types (Figures 6A,B). HTD and MTD shared seven species, LTD and MTD shared five species, and no common fungal species were detected between LTD and HTD. The numbers of unique species identified in LTD, MTD, and HTD were seven, five, and 10, respectively, indicating distinct temperature-dependent characteristics of the fungal communities. According to the culturomics results (Figures 6C,D), eight fungal species were shared among the three Daqu types. HTD and MTD shared two species, LTD and MTD shared eight species, while no common species were detected between LTD and HTD. The numbers of unique species detected by culturomics in LTD, MTD, and HTD were two, three, and four, respectively. Overall, both approaches revealed distinct differences in the fungal community composition across the three Daqu types. Notably, TGS detected a greater number of both shared and unique species compared with culturomics, highlighting its superior resolution in uncovering fungal diversity in Daqu.

## 4 Discussion

This study provides a comprehensive characterization of fungal communities in LTD, MTD, and HTD by integrating culturomics and TGS. Culturomics enabled the isolation of 135 fungal strains, including both yeasts and filamentous fungi, with *S. fibuligera*, *A. chevalieri*, and *L. ramosa* predominant across different Daqu types. In contrast, TGS revealed higher overall fungal richness, identifying 109 ASVs spanning three phyla and 54 species, including several uncultured taxa. Both approaches consistently indicated distinct, temperature-dependent fungal community structures, with *L. ramosa* predominating in LTD and MTD, whereas *T. crustaceus* dominated in HTD. Despite methodological differences, both datasets highlighted the ecological adaptability of several dominant taxa, including *L. ramosa* and *T. crustaceus*. Collectively, these findings demonstrate that integrating culturomics and TGS provides a more complete understanding of fungal diversity and ecological distribution patterns in Baijiu Daqu.

Accurate identification of culturable filamentous fungi is essential for a comprehensive understanding of fungal community composition in Daqu. Although ITS sequencing is widely accepted as the primary fungal barcode (Schoch et al., 2012), it often lacks sufficient resolution to discriminate among closely related species (Wang et al., 2016; Wang X. et al., 2019). In this study, ITS, *β*-tubulin, and CaM gene sequences were combined to improve taxonomic resolution and reduce the risk of misidentification (Figures 2B, Supplementary Figure S2). This multi-locus approach enabled more accurate species-level identification for most isolates. However, some taxa, such as *Aspergillus flavus* and *A. oryzae*, remained indistinguishable even using multi-gene analysis, consistent with previous reports indicating that these two species share highly similar ITS and protein-coding gene sequences (Kusumoto et al., 2000). Notably, *A. flavus* is capable of producing aflatoxins (Klich, 2007), whereas *A. oryzae* has been used for centuries in Asian food fermentation (Tominaga et al., 2006). The close genetic relationship between these species underscores both the taxonomic challenges involved and the importance of rigorous strain-level verification to ensure the safety of Baijiu fermentation.

Although both TGS and culturomics detected dominant fungal taxa, such as *L. ramosa* and *T. crustaceus* (Figures 2C, 3B), notable differences in overall community composition were still observed (Figure 5). These discrepancies primarily arise from fundamental differences in what each method detects. TGS is based on total DNA extracted from environmental samples and therefore detects genetic material derived from viable cells, nonviable cells, and extracellular DNA (Emerson et al., 2017). In contrast, culturomics reflects only those fungal taxa that are metabolically active and capable of growth under defined laboratory cultivation conditions. Previous studies have shown that some microorganisms generally regarded as “culturable” may fail to grow after entering a dormant state (Connon and Giovannoni, 2002), a phenomenon referred to as the “viable but non-culturable” (VBNC) state (Xu et al., 1982). Moreover, the use of fixed culture media and incubation parameters may preferentially enrich specific taxa, thereby underrepresenting the true ecological complexity of fungal communities in Daqu. In addition to methodological bias, ecological filtering during fermentation may further contribute to discrepancies between culturomics and TGS results. A clear example is observed in HTD samples: although yeast-associated ASVs were detected by TGS, no viable yeast strains were recovered through culturomics. This discrepancy is likely attributable to intense thermal stress during HTD fermentation, during which peak temperatures can reach 60–70 °C, exceeding the tolerance limits of most mesophilic yeasts and leading to cellular inactivation. Consequently, yeast cells may remain detectable at the DNA level while failing to grow under standard laboratory cultivation conditions. Despite these discrepancies, the two strategies provide highly complementary insights into fungal community structure. TGS offers a high-resolution overview of fungal diversity at the community level, while culturomics enables the isolation of active and industrially valuable fungal strains from complex environments, thereby providing a critical foundation for subsequent functional and applied research.

The fungal community compositions of the three types of Daqu were obviously differentiated (Figure 3G), with each type exhibiting distinct fungal biomarkers (Figure 4). Temperature is a key environmental determinant shaping fungal diversity in Daqu (Zheng et al., 2015). During Daqu fermentation, internal temperature gradually increases as bioheat accumulates, reaching peak values of 40–50 °C for LTD, 50–60 °C for MTD, and 60–70 °C for HTD (Jin et al., 2017). Subsequently, temperature decreases toward ambient levels as microbial metabolic activity declines and accumulated heat dissipates (Jin et al., 2019, 2020). A reduction in fermentation temperature leads to decline in the relative abundance of thermophilic microorganisms (Xiao et al., 2017). In addition, differences in peak temperature among the three types of Daqu affect moisture evaporation rates, thereby further modifying the ecological niches available for fungal growth. Specifically, the mesophilic fungus *L. ramose* dominated in both LTD and MTD (Figure 3B). This species produces thermostable *α*-amylase and mannanase, which enhance the production efficiency of maltose and mannose, respectively (Zhu et al., 2023). These sugars facilitate the growth of *Pichia* species, thereby contributing to Baijiu flavor development (Cao et al., 2025). In contrast, HTD exhibited selective enrichment of *T. crustaceus* and *A. chevalieri* (Figures 3B, 4). These thermophilic fungi thrive under high temperature and low water activity conditions, while heat-sensitive species are eliminated during fermentation. Consequently, the fungal community structure in HTD becomes increasingly specialized and simplified, resulting in the lowest fungal diversity among the three Daqu types (Figures 3C–F). Owing to its high esterification efficiency and excellent thermostability, *T. crustaceus* is recognized as a key functional fungus responsible for esterase production in HTD (Yang et al., 2025). Meanwhile, *A. chevalieri* secretes multiple enzymes, including amylases, proteases, peptidases, and esterases (Hou et al., 2024), which promote the degradation and utilization of starch, proteins, and ester compounds in raw materials, providing essential precursors for flavor compound formation. Overall, temperature gradients generated by distinct peak temperatures, together with accompanying moisture variations, collectively shaped the ecological differentiation of fungal communities among the three types of Daqu, thereby influencing enzyme activity profiles and metabolic potentials closely associated with Baijiu flavor formation.

Compared with previous studies, the dominant fungal species identified in different types of Daqu in this study showed both consistencies and discrepancies. For example, Han et al. (2025) reported that *L. ramosa* and *P. kudriavzevii* were dominant in LTD from Shanxi Province, while *L. ramosa* predominated in MTD from Sichuan Province. In contrast, Guo et al. (2026) identified *T. aurantiacus* and *Thermomyces lanuginosus* as the predominant fungi in MTD. Moreover, since HTD includes multiple subtypes, *T. crustaceus* and *T. aurantiacus* have been reported as the dominant fungi in black and white HTD, respectively (Cui et al., 2025). These discrepancies relative to our findings may be attributed to differences in the geographical origin of Daqu, local climatic conditions, and fermentation practices. Collectively, such environmental and technological variations impose distinct selective pressures that shape the successional dynamics and dominance patterns of fungal communities in Daqu.

Culturomics enables the high-throughput isolation of cultivable fungi, providing a powerful complement to TGS analyses and expanding the accessible fungal diversity of Daqu. Importantly, the fungal isolates obtained in this study represent valuable microbial resources with potential applications. The amylases produced by the mesophilic fungus *L. ramosa* exhibit high structural stability, allowing sustained enzymatic activity and saccharification efficiency under the variable conditions of industrial fermentation (De Oliveira et al., 2016). Tang et al. (2025) demonstrated that inoculating of Daqu with *S. fibuligera* significantly increased ethyl acetate content without affecting the basic physicochemical parameters of Baijiu, while Li et al. (2020) reported that the addition of aroma-producing yeasts to Daqu reshaped fungal community composition and increased ethyl hexanoate production during fermentation. Collectively, these findings indicate that functional strains can improve the flavor profile of fermented products by modulating microbial community metabolism.

From the perspective of the emerging Qu-omics, this study provides both high-resolution species-level identification and viable fungal isolates, thereby establishing a critical foundation for Qu-omics. Although multi-omics approaches emphasize functional integration, precise species-level resolution and access to cultivable microbial resources remain essential for mechanistic validation. Consequently, the fungal isolates and high-resolution community profiles obtained here provide a critical ecological and biological foundation for future Qu-omics studies integrating metatranscriptomics, proteomics, and metabolomics, ultimately facilitating the rational development and industrial application of functional fungal strains.

## 5 Conclusion

Overall, our findings highlight the fungal diversity and ecological patterns associated with Baijiu Daqu. The integration of TGS and culturomics enabled a comprehensive assessment of fungal diversity across three types of Baijiu Daqu. While TGS provided high-resolution taxonomic profiling, culturomics yielded living isolates that are essential for functional characterization. Both approaches revealed temperature-dependent fungal community patterns and identified key dominant species exhibiting broad ecological adaptability. Collectively, these findings deepen our understanding of fungal ecology in Daqu fermentation and provide a scientific basis for the safe and efficient utilization of functional fungi in Baijiu production.

## Data Availability

The sequencing data generated in this study have been deposited in the Genome Sequence Archive (GSA) under the accession number CRA033565 and are publicly accessible at the GSA database (https://ngdc.cncb.ac.cn/gsa).

## Footnotes

1
https://www.bmgtechno.com

2
http://blast.ncbi.nlm.nih.gov
