# Supplementary material for: Integrative analysis of fungal communities in three types of Baijiu Daqu using third-generation sequencing and culturomics
Source: Front Microbiol. 2026 Jan 14;16:1748163. doi: 10.3389/fmicb.2025.1748163 (PMC12847316; doi:10.3389/fmicb.2025.1748163)
Supplement: Supplementary file 1 [file Supplementary_file_1.docx]

Supplementary Material

# Supplementary Figures


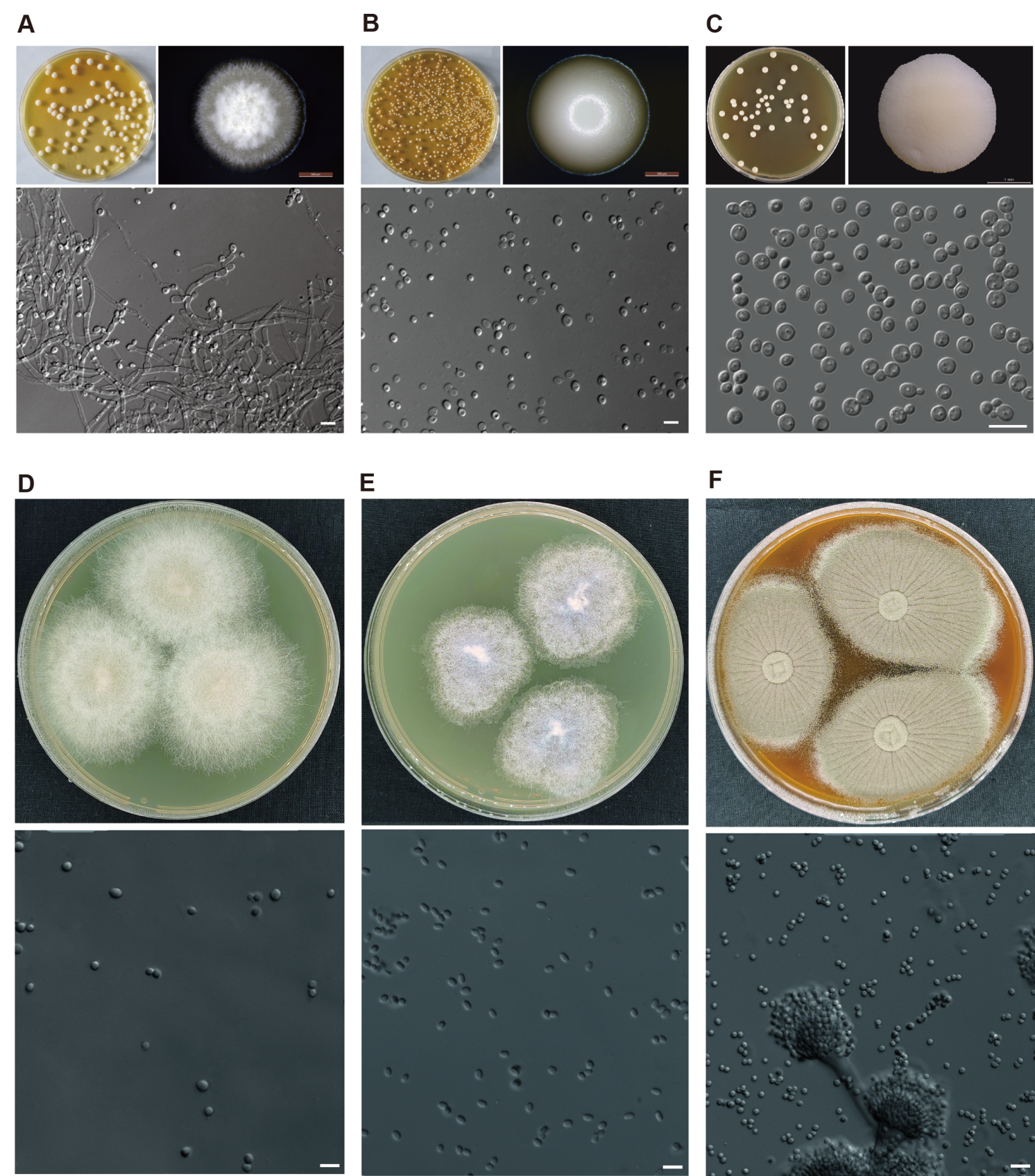


**Supplementary Figure 1.** Colony and microscopic morphologies of the predominant yeasts and filamentous fungi isolated from three types of Daqu using the culturomics. (A) *Saccharomycopsis fibuligera* (B) *Saccharomyces cerevisiae* (C) *Wickerhamomyces anomalus* (D) *Rhizomucor pusillus* (E) *Lichtheimia ramosa* (F) *Aspergillus fumigatus*.


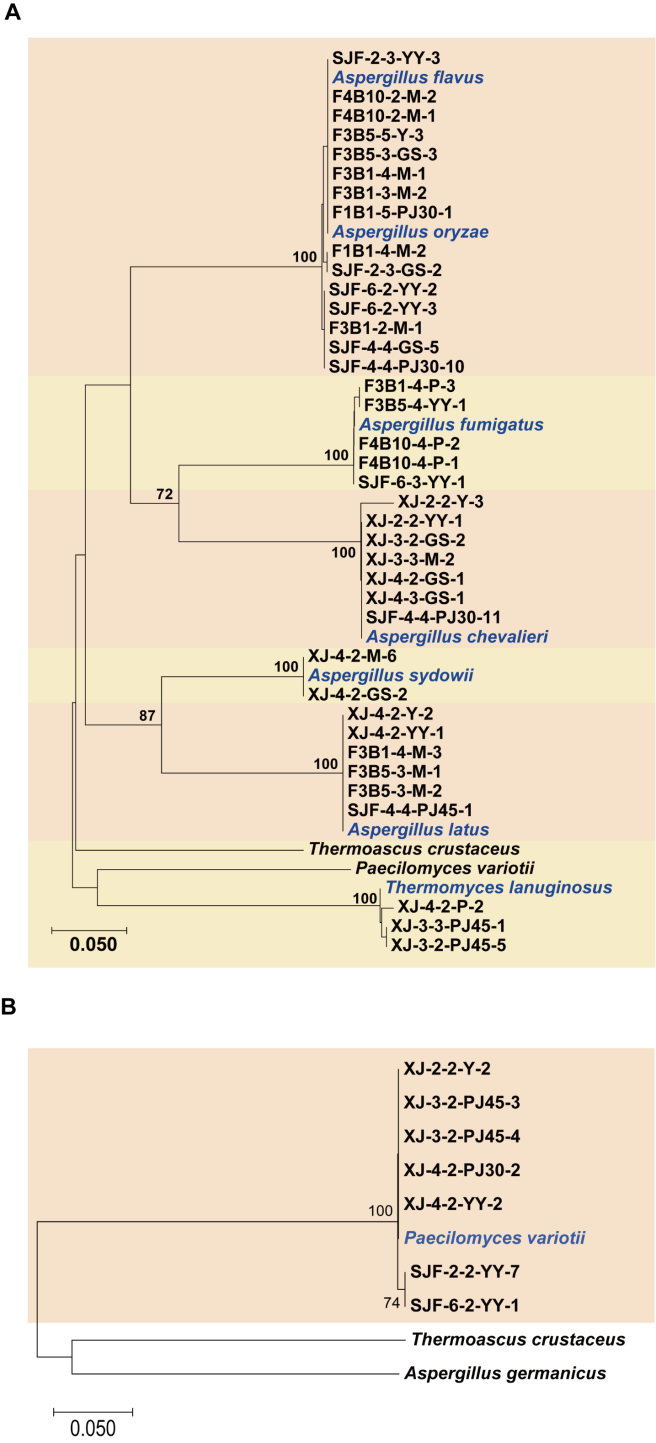


**Supplementary Figure 2.** Phylogenetic trees of cultivable filamentous fungi constructed based on other protein-coding genes. (A) Phylogenetic tree of *Aspergillus* and *Thermomyces* species based on calmodulin (CaM) gene sequences. Species *Thermoascus crustaceus* and *Paecilomyces variotii* are used as the outgroup; (B) Phylogenetic tree of *Paecilomyces* species based on β-tubulin gene sequences. Species *Thermoascus crustaceus* and *Aspergillus germanicus* are used as the outgroup. Bootstrap values ≥70% are shown on the branches of the tree. Bar, 0.05 substitutions per nucleotide position.
